# Hypoxia-induced miR-181a-5p up-regulation reduces epirubicin sensitivity in breast cancer cells through inhibiting EPDR1/TRPC1 to activate PI3K/AKT signaling pathway

**DOI:** 10.1186/s12885-024-11906-6

**Published:** 2024-02-02

**Authors:** Yunwei Zhang, Yunping Guan, Xinyu Zheng, Chenyang Li

**Affiliations:** 1 Department of Breast Surgery, The First Hospital of China Medical University, 155 North Nanjing Street, Shenyang, Liaoning China; 2 Department of Breast Clinic, Shenyang Maternity and Child Health Hosital, No. 20, Yuanjiang Street, Shenyang, Liaoning China; 3 Lab 1, Cancer Institute, The First Hospital of China Medical University, 155 North Nanjing Street, Shenyang, Liaoning China

**Keywords:** Breast cancer, miR-181a-5p, Hypoxia, Epirubicin, EPDR1

## Abstract

Breast carcinoma (BC) ranks as a predominant malignancy and constitutes the second principal cause of mortality among women globally. Epirubicin stands as the drug of choice for BC therapeutics. Nevertheless, the emergence of chemoresistance has significantly curtailed its therapeutic efficacy. The resistance mechanisms to Epirubicin remain not entirely elucidated, yet they are conjectured to stem from diminished tumor vascular perfusion and resultant hypoxia consequent to Epirubicin administration. In our investigation, we meticulously scrutinized the Gene Expression Omnibus database for EPDR1, a gene implicated in hypoxia and Epirubicin resistance in BC. Subsequently, we delineated the impact of EPDR1 on cellular proliferation, motility, invasive capabilities, and interstitial-related proteins in BC cells, employing methodologies such as the CCK-8 assay, Transwell assay, and western blot analysis. Our research further unveiled that hypoxia-induced miR-181a-5p orchestrates the regulation of BC cell duplication, migration, invasion, and interstitial-related protein expression via modulation of EPDR1. In addition, we identified TRPC1, a gene associated with EPDR1 expression in BC, and substantiated that EPDR1 influences BC cellular dynamics through TRPC1-mediated modulation of the PI3K/AKT signaling cascade. Our findings underscore the pivotal role of EPDR1 in the development of BC. EPDR1 was found to be expressed at subdued levels in BC tissues, Epirubicin-resistant BC cells, and hypoxic BC cells. The overexpression of EPDR1 curtailed BC cell proliferation, motility, invasiveness, and the expression of interstitial-related proteins. At a mechanistic level, the overexpression of hypoxia-induced miR-181a-5p was observed to inhibit the EPDR1/TRPC1 axis, thereby activating the PI3K/AKT signaling pathway and diminishing the sensitivity to Epirubicin in BC cells. In summation, our study demonstrates that the augmentation of hypoxia-induced miR-181a-5p diminishes Epirubicin sensitivity in BC cells by attenuating EPDR1/TRPC1 expression, thereby invigorating the PI3K/AKT signaling pathway. This exposition offers a theoretical foundation for the application of Epirubicin in BC therapy, marking a significant contribution to the existing body of oncological literature.

## Introduction

Breast cancer ranks as the second prevalent type of cancer among women. According to cancer statistics, new cases of BC account for about one-quarter of all cancer cases in women are malignancies [1]. The main treatments for BC include surgery, radiotherapy, chemotherapy, and immunotherapy [2]. Almost all patients with primary BC have a good prognosis after treatment, featuring a general five-year survival rate of 99% [1]. However, distal metastases occur in BC and the 5-year survival rate is only 26% in patients with distant metastases [3]. Chemotherapy is a traditional and necessary treatment to reduce mortality in metastatic BC patients [4]. Drug resistance is a major cause of chemotherapy failure [5]. BC is resistant to various drugs through the promotion of drug efflux pumps, alteration of drug targets, activation of bypass signaling pathways, maintenance of stemness, and dysregulation of immune responses [6]. Research on chemoresistance in BC is necessary to develop new biomarkers and therapeutic targets to predict therapy or improve clinical outcomes.

Anthracyclines are well-known as effective anti-tumor agents, including BC, leukemia, lymphoma and sarcoma, which can significantly improve cancer survival. Anthracyclines are the standard treatment for BC [7]. Epirubicin is the most commonly used anthracycline in BC treatment [8, 9]. However, drug resistance limits its efficacy [10]. Qiong et al. demonstrated that Orosomucoid 1(ORM1) promotes epirubicin resistance in BC cells by regulating MMP-2 and MMP-9 expression and activating the AKT/ERK signaling pathway [11]. Vitale et al. demonstrated that knockdown of uridine diphosphate glucose dehydrogenase induced epirubicin resistance in triple-negative BC cells, which may be associated with increased cellular hyaluronan levels, deposition and catabolism [10]. Ma et al. reported that CCL5 promoted EMT and induced epirubicin resistance in BC cells [12]. Wang et al. demonstrated that lnc005620 promoted progression and enhanced epirubicin resistance in triple-negative BC cells by upregulating ITGB1 expression [13]. However, the detailed mechanisms underlying epirubicin resistance in BC cells are not yet fully understood. Further investigation into epirubicin resistance may provide a theoretical basis for its application.

Hypoxia is a common feature in both primary and metastatic BC. The level of HIF-1α in cancerous tissues is linked to unfavorable outcomes and resistance to chemotherapy [14]. Hypoxia-induced HIF-1α activation regulates drug resistance in BC through different pathways including increased expression of efflux pumps, upregulation of autophagy, inhibition of apoptosis, maintenance of stemness and metabolic reprogramming [15]. Milani et al. identified hypoxia-related biomarkers as predictors of epirubicin resistance in BC [16]. Additionally, anthracyclines such as epirubicin reduce blood flow and induce tumor hypoxia-associated growth factors, thereby reducing the therapeutic effect [16]. Thus, epirubicin induced hypoxia in BC, and hypoxia promoted epirubicin resistance in BC. However, the detailed mechanisms require further investigation.

Therefore, differentially expressed genes (DEGs) were screened in breast cancer tissue and normal tissue, epirubicin-resistant and parental breast cancer cells, and hypoxic and normoxic treated breast cancer cells relying on data from the gene expression omnibus database. Therefore, we investigated the mechanism by which hypoxia induced epirubicin resistance in BC cells. This study provides a new molecular mechanism to reverse epirubicin resistance in BC and identifies an effective therapeutic agent for BC.

## Materials and methods

### Bioinformatics analysis

GSE42568, GSE54326, GSE111246 and GSE49999 datasets were obtained from the GEO database. The GSE42568 dataset included 104 BC and 17 normal breast tissue samples; the GSE54326 dataset included three epirubicin-resistant MCF-7 cells and three parental MCF-7 cell samples; the GSE111246 dataset included three 20% oxygen-cultured MCF-7 cells and three 0.5% oxygen-cultured MCF-7 cell samples; the GSE49999 dataset included three 20% oxygen-cultured MCF-7 cells and three 0.1% oxygen-cultured MCF-7 cell samples. Genes with differential expression in different datasets was analyzed using GEO2R. Differentially expressed gene transcripts were considered significant at cutoff values of|*logFC*| > 1 and FDR < *0.05*. Potential binding sites of miRNAs to mRNA 3’-UTRs were predicted using the ENCORI online database and miRNA expression in BC tissues and its association with the overall survival of breast cancer patients were also analyzed. Genes associated with EPDR1 expression in TCGA database were analyzed using the R software package (version 2.15.3). EPDR1-related genes expressed in BC tissues were analyzed using GEPIA. The correlation between related gene expression and overall survival was analyzed using Kaplan-Meier Plotter.

### Sample collection

The research received approval from the Institutional Review Board and Ethics Committee at The First Hospital of China Medical University, every participant provided informed consent. The study involved collecting and preserving 24 pairs of BC and adjacent non-cancerous tissues at -80 °C. Prior to undergoing surgery, none of patients had been treated with chemotherapy or radiotherapy.

### Cell cultivation and transfection

MCF-7 was obtain from Wuhan Pu-nuo-sai Life Technology Co., Ltd.(Wuhan, China). The epirubicin-resistant cell line, MCF-7/EPI, was prepared by serially increasing concentrations of *epirubicin* for selection. Briefly, 0.5 µg/mL of epirubicin was added to cultured cells and incubated for 24 h. The medium was then changed. Cell was sub-cultured as the confluence was about 80%. After reaching passage 2, 1 µg/mL epirubicin was added, and the above steps were repeated. Epirubicin concentrations of 0.5, 1, 1.5, 2, 2.5, 3, 3.5, 4, 5, 6, and 7 µg/mL were added successively to obtain MCF7/EPI cells with IC50 value of 56.57 µg/mL and a drug resistance index of 13.15. Cells were grown in DMEM/high glucose medium supplemented with 10% FBS, containing 1% double-antibiotics, maintained at 37 °C, 5% CO_2_ atmosphere. A three-gas incubator was used to prepare the hypoxic cell model in vitro. The hypoxic conditions were 0.1% O_2_, 5% CO_2_, and 94.9% N_2_. The lentivirus and control lentivirus used to overexpress EPDR1 were purchased from Shanghai Genechem Medical Technology Co., Ltd. The vector GV341 was resistant to puromycin. Transfection of cells was meticulously conducted following the guidelines provided by the manufacturer. Suzhou GenePharma Co., Ltd., based in Suzhou, China, synthesized the miR-181a-5p mimics, inhibitors, and their respective controls. For the transfections, the final concentration of miR-181a-5p mimics or inhibitors and the controls was set at 100 nM.

### Quantitative real-time PCR RT-qPCR assay

Whole RNA was extracted using RNAiso Plus from TaKaRa, Japan. After reverse transcribed into complementary DNA using the PrimeScript RT Master Mix(TaKaRa), To detect the expression levels of miR-181a-5p and EPDR1 mRNA, TB Green® Premix Ex Taq™ II, provided by TaKaRa, was used. GAPDH and U6 served as the internal controls. The relative expression levels were determined using 2^−ΔΔCt^ method. Below are the primers that were employed in the assay.

miR-181a-5p-F: 5’-GCCGAACATTCAACGCTGTCG-3’;

miR-181a-5p-R: 5’-GTGCAGGGTCCGAGGT-3’;

U6-F: 5’-CTCGCTTCGGCAGCACA-3’;

U6-R: 5’-AACGCTTCACGAATTTGCGT-3’;

EPDR1-F: 5’-TGAAACCTGGATTGGCATCT ATAC-3’;

EPDR1-R: 5’-TGTAGTTTATGGTAAAGGTTTCCTG-3’;

GAPDH-F: 5’-ATGGAAATCCCATCACCATCTT-3’;

GAPDH-R: 5’-CGCCCCACTTGATTTTG G-3’.

### Western blot analysis

Total proteins were carefully extracted from both tissue and cellular samples using RIPA lysis buffer. The concentrations were then quantified using the BCA method. These proteins were separated by SDS-PAGE and transferred onto PVDF membranes. Following the transfer, the membranes were blocked with 5% skim milk and then incubated with primary antibodies targeting EPDR1, N-cadherin, Vimentin, Snail, TRPC1, PI3K, phospho-AKT (Ser473), AKT, and GAPDH overnight at 4 °C. For detection, horseradish peroxidase-conjugated secondary antibodies, either goat anti-rabbit or anti-mouse, were used. Signal detection was conducted using BeyoECL Plus and a gel imaging system. ImageJ software was utilized for the quantitative analysis of the image gray values.

### Counting kit-8 (CCK-8) assay

Cells were carefully plated in 96-well plates, with a seeding density of 2 × 10³/well, following the manufacturer’s instructions precisely. At specific time intervals, medium was replaced with CCK-8 reagent. Cells were then incubated at 37 °C for 1 h. After incubation, the absorbance was measured at 450 nm using an advanced microplate reader.

### Invasion and migration assays

A dual approach was utilized for cell detection. Cells designated for migration analysis were transferred to the upper chamber of a noncoated membrane chamber, while those for invasion analysis were placed in a chamber coated with Matrigel, both set in DMEM supplemented with 5% fetal calf serum. The lower chamber was filled with DMEM containing 20% fetal calf serum, serving as a potent chemoattractant. Following a 24-hour incubation period, cells that did not invade through the membrane were carefully removed from the upper well. The cells that successfully invaded were fixed using 4% paraformaldehyde and subsequently stained with crystal violet for clear visualization. The final results were meticulously observed and captured using an optical microscope, allowing for a detailed analysis of cellular invasive and migratory capabilities.

### Dual luciferase assay

The luciferase gene plasmids were purchased from Suzhou GenePharma Co., Ltd. When cell density reached approximately 50%, the firefly luciferase plasmid and Renilla luciferase plasmid were transfected. After 24 h of cultivation, firefly and Renilla luciferase activity was calculated using the Dual-Glo Luciferase Assay System (Promega, USA) and GloMax® 20/20 Luminometer (Promega, USA). Relative luciferase activity = firefly luciferase activity / Renilla luciferase activity ×100%.

### AGO2-RIP assay

RIP was performed using the Magna RIP RNA Binding Protein Immunoprecipitation Kit (Millipore, Billerica, MA, USA).Immunoprecipitated RNA was then extracted and miR-181a-5p and EPDR1 mRNA enrichment was detected by RT-qPCR.

### Animal experiment

Nude mice, female, were obtained from Beijing Biocytogen Co., Ltd. (Beijing, China). The animal procedures were approved by the Animal Welfare and Ethical Committee of China Medical University, China. Nude mice were randomly divided into two groups with 5 mice each. Stably overexpressed EPDR1 or overexpressed control MCF7 cells (1 × 10^7^) in Matrigel (BD Biosciences, USA) were injected into the right flanks of mice to form xenograft tumors. The average volume of the tumor was measured three times every 3 days. The tumor volumes were calculated using the following formula: (L × W^2^)/2.

Meanwhile, the nude mice were divided into two groups with 3 mice each. The 1 × 10^7^ MCF7 cells statically overexpressing EPDR1 or overexpressing control cells were injected into nude mice through the tail vein to establish the in vivo metastasis model. Metastasis was observed by bioluminescence imaging 30 days after injection. Lung tissues of nude mice were subjected to gross morphological observation and HE staining.

### Statistical analysis

The outcomes of this study are articulated as the mean ± standard deviation (S.D.), derived from three independent experimental replicates, barring any specific stipulations. The entirety of the data underwent a rigorous analytical process utilizing SPSS software, version 22.0 (IBM, USA), ensuring statistical robustness and accuracy. To discern and evaluate the differences between distinct experimental groups, the Student’s t-test was employed as a reliable statistical tool. Additionally, for comprehensive comparisons encompassing multiple groups, a one-way analysis of variance (ANOVA) was conducted, accompanied by Tukey’s multiple comparison post hoc test. This meticulous approach ensured a thorough and nuanced analysis of the data. In this context, a *p*-value threshold of less than 0.05 was established as the criterion for statistical significance, thereby underscoring findings of substantial relevance in the studied phenomena.

## Results

### EPDR1 was lowly expressed in BC tissues, epirubicin-resistant BC cells, and hypoxia-treated BC cells

**Fig. 1:**
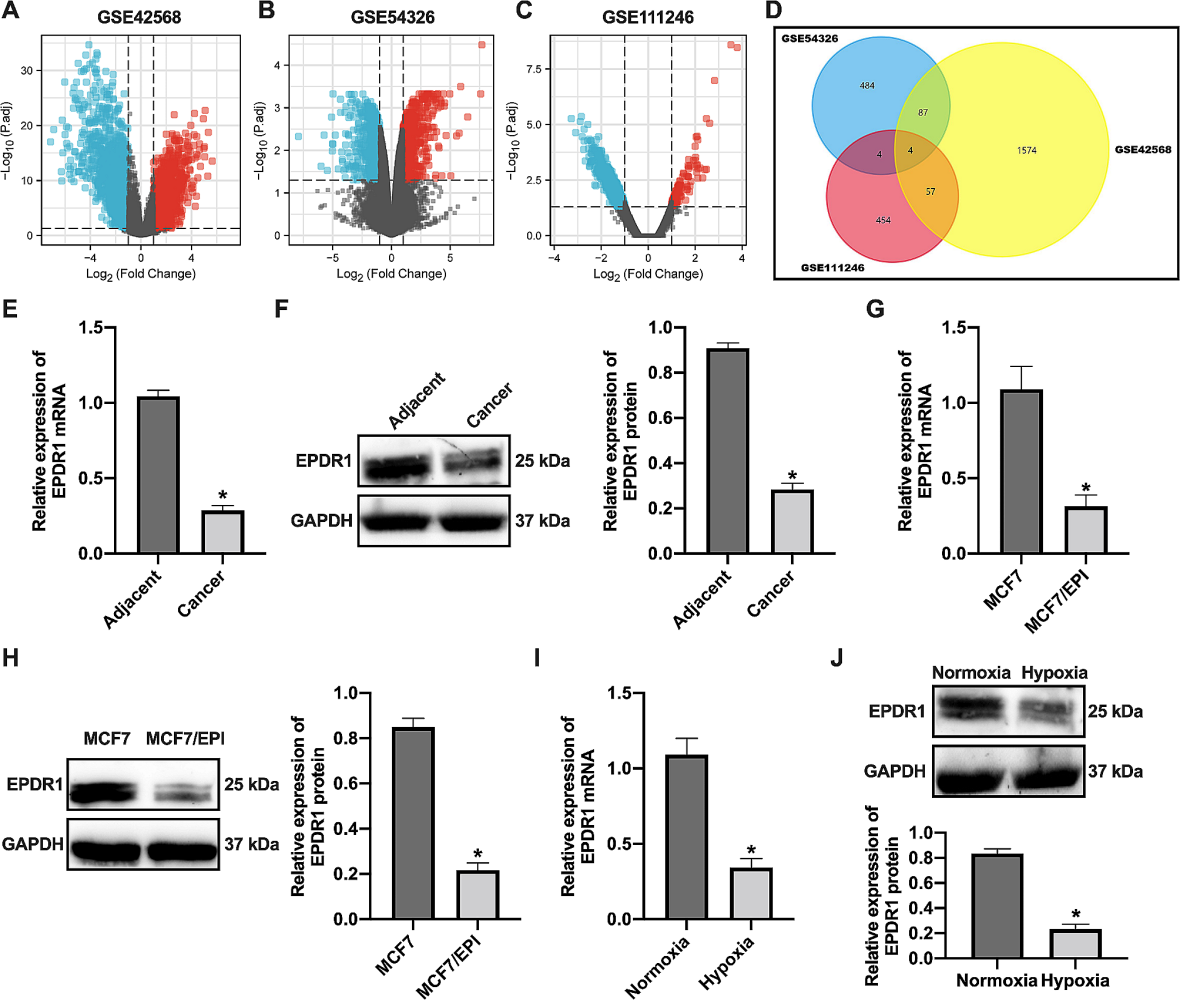
EPDR1 lowly expressed in BC tissues, epirubicin-resistant BC cells and hypoxia-treated BC cells. (**A**) DEGs of GSE42568 dataset were analyzed by GEO2R. (**B**) DEGs of GSE54326 dataset were analyzed by GEO2R. (**C**) DEGs in the GSE111246 dataset were analyzed by GEO2R online analysis software. (**D**) The genes to be lowly expression in BC tissues, epirubicin-resistant BC cells and hypoxia-treated BC cells were analyzed by Venn diagram analysis. (**E**) The expression of EPDR1 mRNA in BC and paracancer tissues was examined by RT-qPCR; *, vs. Adjacent group, *p* < 0.05. (**F**) The expression of EPDR1 protein in BC and paracancer tissues was examined by Western blot; *, vs. Adjacent group, *p* < 0.05. (**G**) The expression of EPDR1 mRNA in epirubicin-resistant and parental MCF-7 cells was examined by RT-qPCR; *, vs. MCF7 group, *p* < 0.05. (**H**) The expression of EPDR1 protein in epirubicin-resistant and parental MCF-7 cells was examined by Western blot; *, vs. MCF7 group, *p* < 0.05. (**I**) The expression of EPDR1 mRNA in hypoxic and normoxic treated MCF-7 cells was examined by RT-qPCR; *, vs. Normoxia group, *p* < 0.05. (**J**) The expression of EPDR1 protein in hypoxic and normoxic treated MCF-7 cells was examined by western blot assay; *, vs. Normoxia group, *p* < 0.05

EPDR1 (Fig. 1D) was expressed lowly in BC tissues, epirubicin-resistant BC cells, and hypoxia-treated BC cells (Fig. 1A-D). Consistent with the results of the bioinformatic analysis, EPDR1 was expressed at low levels in BC tissues (Fig. 1E-F), epirubicin-resistant BC cells (Fig. 1G-H), and hypoxia-treated BC cells (Fig. 1I-J).

### Overexpression of EPDR1 inhibits the malignant progression of BC

**Fig. 2:**
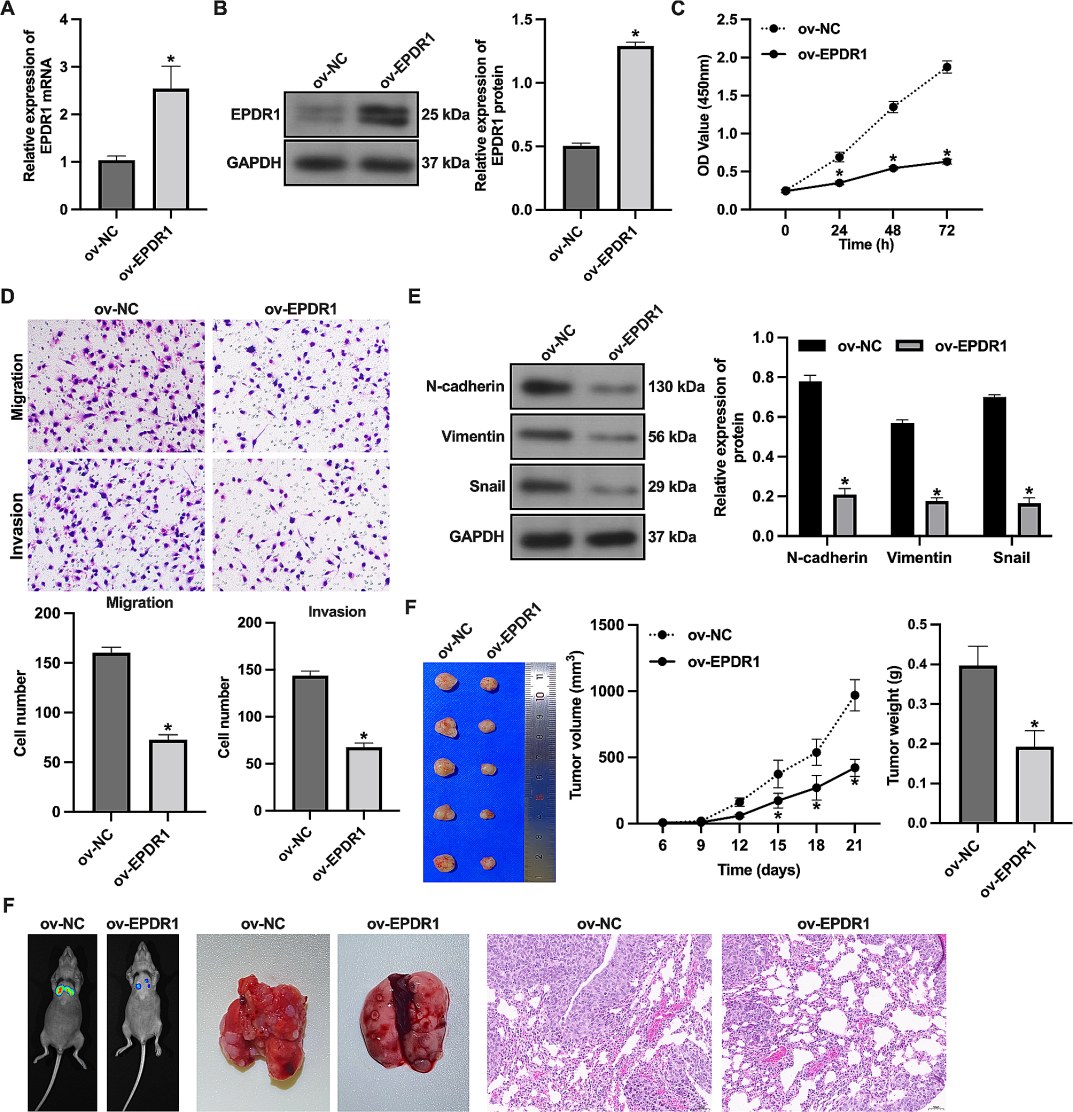
Overexpression of EPDR1 inhibits malignant progression of BC. The MCF-7 cells stably overexpressing EPDR1. (**A**) The expression of EPDR1 mRNA was examined by RT-qPCR. (**B**) The expression of EPDR1 protein was examined by western blot assay. (**C**) The proliferation of cells was examined by CCK-8 assay. (**D**) The migration and invasion of cells was examined by Transwell assay. (**E**) The expression of EMT-related protein was examined by western blot assay. (**F**) Tumor volume and weight. (**G**) Tumor metastasis was observed by bioluminescence imaging, lung tissue gross observation, and HE staining. *, vs. ov-NC group, *p* < 0.05

Then we established MCF-7 cells stably overexpressing EPDR1 (Fig. 2A-B). Overexpression of EPDR1 reduced the proliferation of MCF-7 cells (Fig. 2C), and inhibited their migration and invasion (Fig. 2D). Overexpression of EPDR1 also inhibited N-cadherin, Vimentin, and Snail protein expression (Fig. 2E). Similarly, in the nude mouse model, overexpression of EPDR1 inhibited tumor growth (Fig. 2F) and metastasis (Fig. 2G). These results suggested that EPDR1 inhibits BC cell proliferation, migration, invasion, and interstitial-related protein expression in vitro as well as tumor growth and metastasis in vivo.

### Hypoxia down-regulates EPDR1 expression in BC cells through up-regulation of miR-181a-5p

**Fig. 3:**
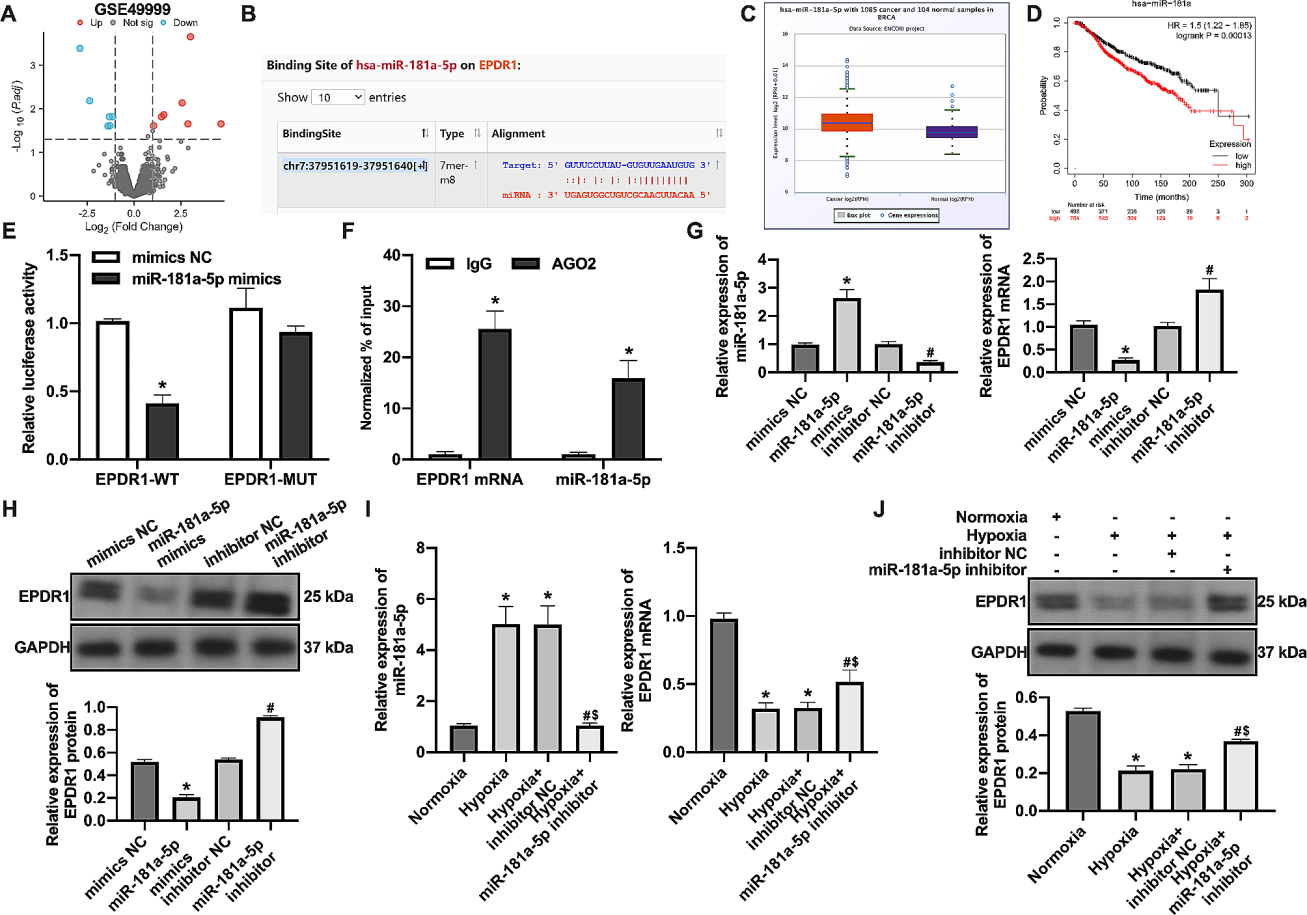
Hypoxia down-regulates EPDR1 expression in BC cells through up-regulation of miR-181a-5p. (**A**) The differentially expressed genes in the GSE49999 dataset were analyzed by GEO2R online analysis software. (**B**) The potential binding site was between miR-181a-5p and EPDR1 mRNA. (**C**) The expression of miR-181a-5p in BC was analyzed by ENCORI. (**D**) The relation between miR-181a-5p and BC prognosis was analyzed by Kaplan-Meier Plotter. (**E**) The binding of miR-181a-5p to EPDR1 mRNA was confirmed by dual luciferase reporter gene assays; *, vs. mimics NC group, *p* < 0.05. (**F**) The binding of miR-181a-5p to EPDR1 mRNA was confirmed by AGO2-RIP; *, vs. IgG group, *p* < 0.05. Transfection of miR-181a-5p mimics and inhibitor for BC cells, (**G**) the expression of miR-181a-5p and EPDR1 mRNA was examined by RT-qPCR; (**H**) the expression of EPDR1 protein was examined by western blot assay; *, vs. mimics NC group, *p* < 0.05; #, vs. inhibitor NC group, *p* < 0.05. Transfected hypoxia-treated MCF-7 cells with miR-181a-5p inhibitor, (**I**) the expression of miR-181a-5p and EPDR1 mRNA was examined by RT-qPCR; (**J**) the expression of EPDR1 protein was examined by western blot assay; *, vs. Normoxia group, *p* < 0.05; #, vs. Hypoxia group, *p* < 0.05; $, vs. Hypoxia + inhibitor NC group, *p* < 0.05

Hypoxia-induced dysregulation of miRNA expression can regulate the expression of downstream target genes [17]. We screened for upregulated miRNAs under hypoxia (Fig. 3A). The miR-181a-5p was predicted to be a potential binding site for EPDR1 mRNA (Fig. 3B), which is highly expressed in BC (Fig. 3C) and has a poor prognosis in BC patients with high expression (Fig. 3D). Binding of miR-181a-5p to EPDR1 mRNA was confirmed (Fig. 3E-F). Transfection with the miR-181a-5p mimics inhibited EPDR1 expression. In contrast, transfection with miR-181a-5p inhibitor promoted EPDR1 expression (Fig. 3G-H). Inhibition of miR-181a-5p expression increased EPDR1 expression under hypoxia (Fig. 3I-J). These results suggest that hypoxia down-regulates EPDR1 expression in BC cells by upregulating miR-181a-5p.

### Hypoxia reduces epirubicin sensitivity in BC cells through miR-181a-5p/EPDR1

**Fig. 4:**
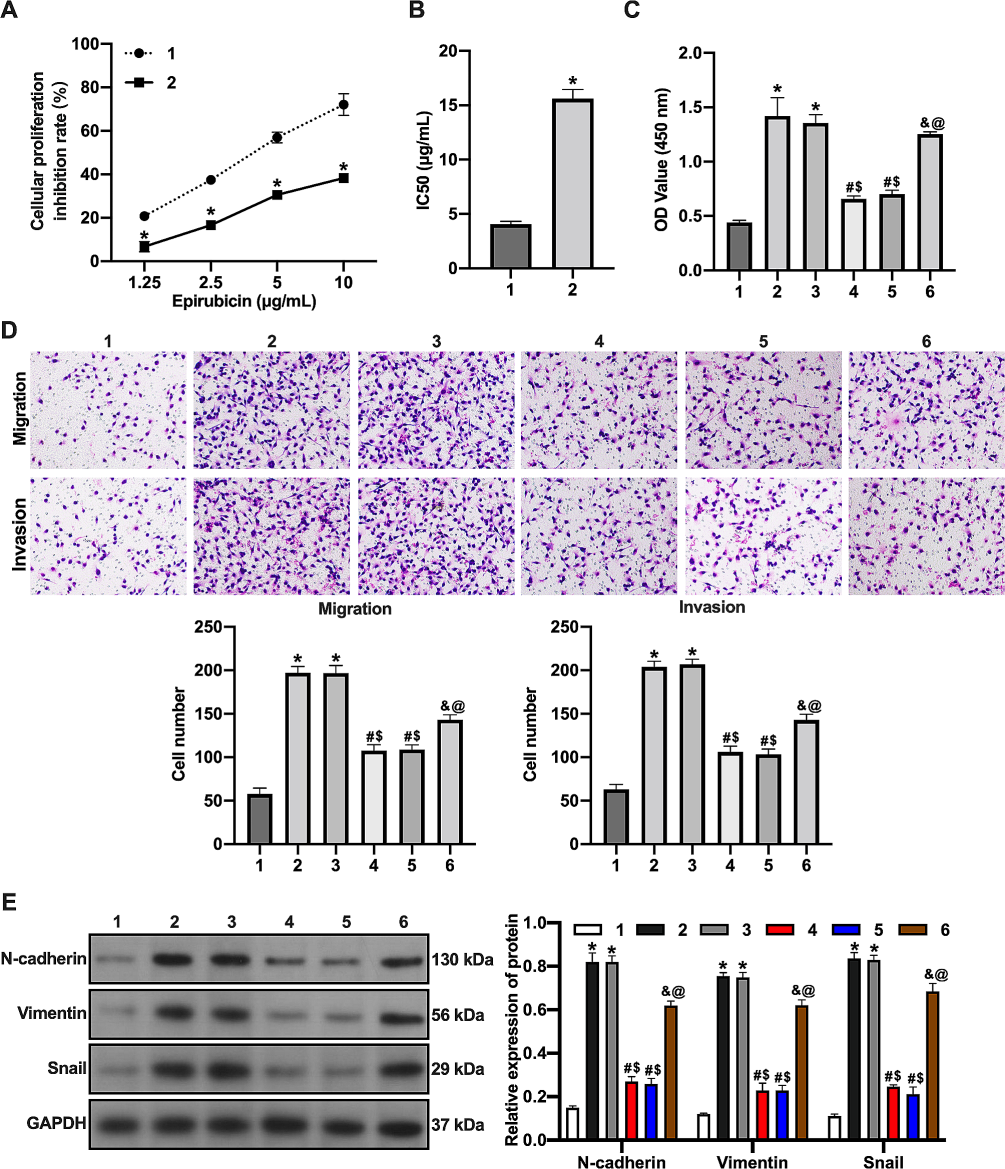
Hypoxia reduces epirubicin sensitivity in BC cells through miR-181a-5p/EPDR1. (**A**) MCF-7 cells in normoxic and hypoxic environments and treated with different concentrations of epirubicin to detect cell activity by CCK-8 assay. (**B**) The IC50 values. (**C**) CCK-8 assay. (**D**) The migration and invasion of cells were examined by Transwell assay. (**E**) The expression of EMT-related protein was examined by western blot assay. 1, Normoxia group; 2, hypoxia group; 3, hypoxia + inhibitor NC group; 4, hypoxia + miR-181a-5p inhibitor group; 5, hypoxia + miR-181a-5p inhibitor + si-NC group; 6, hypoxia + miR-181a-5p inhibitor + si-EPDR1 group. *, vs. Normoxia group, *p* < 0.05; #, vs. Hypoxia group, *p* < 0.05; $, vs. Hypoxia + inhibitor NC group, *p* < 0.05; &, vs. Hypoxia + miR-181a-5p inhibitor group, *p* < 0.05; @, vs. Hypoxia + miR-181a-5p inhibitor + si-NC group, *p* < 0.05

We first cultured MCF-7 cells in normoxic and hypoxic environments and treated them with different epirubicin concentrations to detect cell activity. The results showed that the inhibition rate of MCF-7 cells under hypoxia was lower than that under normoxia (Fig. 4A). In addition, the IC50 values of hypoxia-treated MCF-7 cells were higher than those of the normoxic cells (Fig. 4B). Hypoxia increased proliferation in epirubicin-treated MCF-7 cells, and transfection of miR-181a-5p inhibitor down-regulated hypoxia-induced proliferation activity in epirubicin-treated MCF-7 cells. Silencing of EPDR1 reversed the inhibitory effect of transfection with the miR-181a-5p inhibitor on hypoxia-induced proliferation activity in epirubicin-treated MCF-7 cells (Fig. 4C). Similarly, hypoxia increased the migratory and invasive capacity of epirubicin-treated MCF-7 cells, and transfection with miR-181a-5p inhibitor suppressed the migratory and invasive capacity of hypoxia-induced epirubicin-treated MCF-7 cells, and silencing EPDR1 reversed the effect of transfection with miR-181a-5p inhibitor on hypoxia-induced; The inhibition of the migratory and invasive abilities of epirubicin-treated MCF-7 cells was reversed by silencing EPDR1 (Fig. 4D). Moreover, hypoxia increased N-cadherin, Vimentin, and Snail levels in epirubicin-treated MCF-7 cells, and transfection with miR-181a-5p inhibitor down-regulated the expression of N-cadherin, Vimentin, and Snail under hypoxic conditions. The silencing of EPDR1 reversed the inhibitory effect of transfection with the miR-181a-5p inhibitor on hypoxia-induced N-cadherin, Vimentin and Snail protein expression levels in epirubicin-treated MCF-7 cells (Fig. 4E). These results suggest that hypoxia reduces epirubicin sensitivity of BC cells by inducing miR-181a-5p levels to inhibit EPDR1 expression.

### EPDR1 inhibits the PI3K/AKT signaling pathway in BC epirubicin-resistant cells through the upregulation of TRPC1

**Fig. 5:**
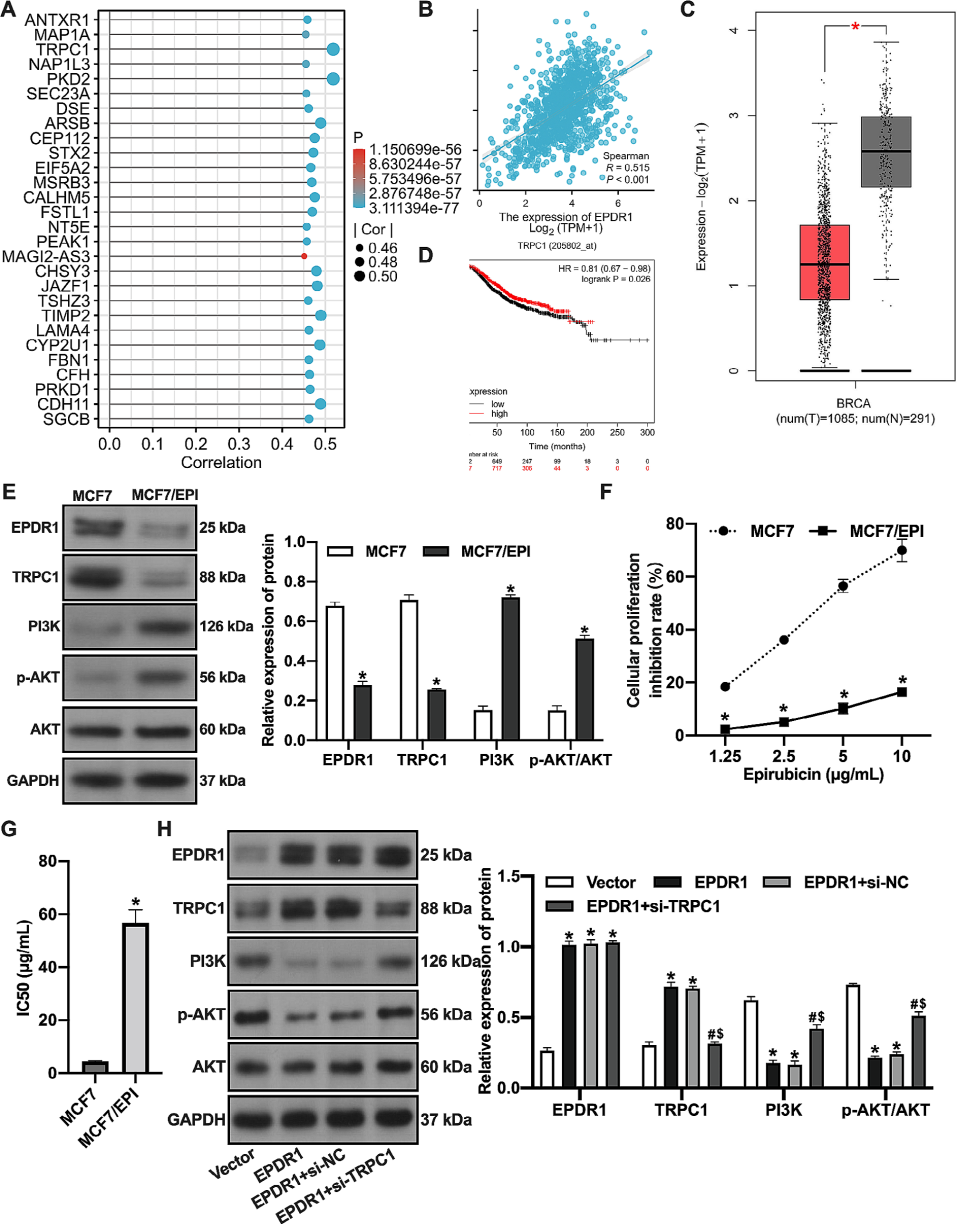
EPDR1 inhibits PI3K/AKT signaling pathway in BC epirubicin-resistant cells through upregulation of TRPC1. (**A**) Genes related to EPDR1 expression in breast cancer were analyzed through bioinformatics. (**B**) TRPC1 was positively correlated with EPDR1 expression. (**C**) GEPIA analysis showed that TRPC1 was lowly expressed in BC tissues. (**D**) Kaplan-Meier Plotter analysis. (**E**) The expression of TRPC1, PI3K and p-AKT were examined by western blot assay. *, vs. MCF7 group, *p* < 0.05. (**F**) The inhibition rates of epirubicin-treated MCF-7 and MCF-7/EPI cells were examined by CCK-8 assay. *, vs. MCF7 group, *p* < 0.05. (**G**) IC50 values; *, vs. MCF7 group, *p* < 0.05. (**H**) The expression of TRPC1, PI3K and p-AKT of MCF-7/EPI cells overexpressing EPDR1 or silencing TRPC1. *, vs. Vector group, *p* < 0.05; #, vs. EPDR1 group, *p* < 0.05; $, vs. EPDR1 + si-NC group, *p* < 0.05

We further investigated the mechanism by which EPDR1 increased epirubicin sensitivity in BC cells. It was found that TRPC1 was positively correlated with EPDR1 expression (Fig. 5A-B). TRPC1 was lowly expressed in BC tissues (Fig. 5C) and BC patients with high TRPC1 expression had better overall survival (Fig. 5D). It has been shown that TRPC1 inhibits BC cell proliferation and migration by suppressing the PI3K/AKT pathway [18]. The activation of the PI3K/AKT pathway is one of the mechanisms by which epirubicin resistance develops in BC cells [19]. Therefore, we verified whether EPDR1 inhibits the PI3K/AKT signaling pathway through upregulation of TRPC1 expression, leading to increasing epirubicin sensitivity in BC cells. An epirubicin-resistant BC MCF-7 cell line (MCF-7/EPI) was established, and MCF-7/EPI cells showed significantly lower EPDR1 levels and TRPC1 levels and remarkably higher PI3K and p-AKT protein expression levels compared to parental cells (Fig. 5E). In addition, the inhibition rate of epirubicin-treated MCF-7/EPI cells was reduced (Fig. 5F), and the IC50 value was increased (Fig. 5G). Overexpression of EPDR1 increased TRPC1 expression and decreased PI3K and p-AKT levels in MCF-7/EPI cells. The TRPC1 silencing increased PI3K and p-AKT protein expression in MCF-7/EPI cells overexpressing EPDR1 (Fig. 5H). These results suggest that EPDR1 inhibits PI3K/AKT activation in BC epirubicin-resistant cells through upregulating TRPC1.

### EPDR1/TRPC1 promotes epirubicin sensitivity in BC cells via inhibiting PI3K/AKT signaling pathway

**Fig. 6:**
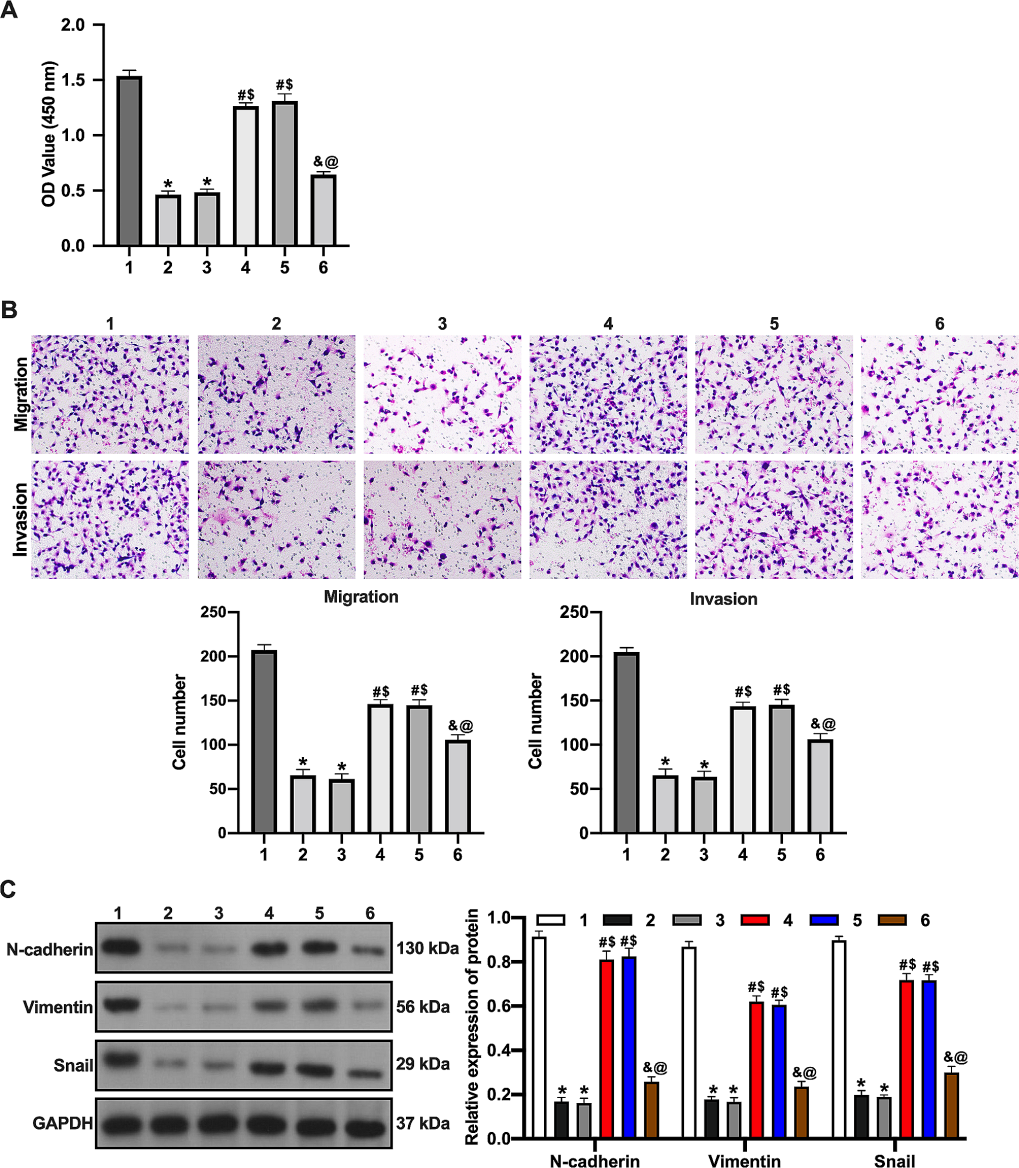
EPDR1/TRPC1 promotes epirubicin sensitivity in BC cells via inhibiting PI3K/AKT signaling pathway. (**A**) The proliferation of cells was examined by CCK-8 assay. (**B**) The migration and invasion of cells was examined by Transwell assay. (**C**) The expression of EMT-related protein was examined by western blot assay. 1, Vector group; 2, EPDR1 group; 3, EPDR1 + si-NC group; 4, EPDR1 + si-TRPC1 group; 5, EPDR1 + si-TRPC1 + DMSO group; 6, EPDR1 + si-TRPC1 + LY294002 group. *, vs. Vector group, *p* < 0.05; #, vs. EPDR1 group, *p* < 0.05; $, vs. EPDR1 + si-NC group, *p* < 0.05; &, vs. EPDR1 + si-TRPC1 group group, *p* < 0.05; @, vs. EPDR1 + si-TRPC1 + DMSO group, *p* < 0.05

Overexpression of EPDR1 decreased proliferation, migration, and invasion ability of epirubicin-treated MCF-7/EPI cells. TRPC1 silencing increased the proliferation of epirubicin-treated cells by overexpressing EPDR1. The administration of PI3K inhibitors reversed the effects of silencing TRPC1 on the proliferation and metastasis of MCF-7/EPI cells treated with epirubicin overexpressing EPDR1 (Fig. 6A-B). Furthermore, overexpression of EPDR1 decreased N-cadherin, Vimentin, and Snail levels in epirubicin-treated MCF-7/EPI cells. TRPC1 silencing increased N-cadherin, Vimentin, and Snail levels in epirubicin-treated MCF-7/EPI cells containing overexpressing EPDR1. The administration of PI3K inhibitor reversed the effect of TRPC1 silencing on N-cadherin, Vimentin and Snail levels in epirubicin-treated MCF-7/EPI cells (Fig. 6C). These results suggest that EPDR1/TRPC1 increases epirubicin sensitivity in BC cells through inhibiting the PI3K/AKT signaling pathway.

## Discussion

We analyzed differentially expressed genes in the GSE42568, GSE54326, and GSE111246 datasets and demonstrated that EPDR1 was lowly expressed in all three datasets, i.e. EPDR1 was lowly expressed in BC tissues, epirubicin-resistant BC cells, and hypoxic BC cells. EPDR1 is a member of the ependymin-related (EPDR) family of proteins, sharing conserved amino acids with fish ventricular tubulin and is a type II transmembrane protein that plays a key role in cell adhesion [20]. In 2001, Nimmrich et al. first identified that EPDR1 is highly expressed in colorectal cancer (CRC) cells [21]. Another study exhibited that EPDR1 promotes the proliferation, migration, invasion, and adhesion of CRC cells to type I collagen fibers [22]. Similarly, Chu et al. reported that EPDR1 promoted the invasion of CRC cells [23]. Yang et al. demonstrated that EPDR1 was highly expressed in bladder cancer tissues and associated with TNM stage and the overall survival rate, which is a prognostic biomarker for bladder cancer [24]. Chen et al. found that EPDR1 was highly expressed in hepatocellular tissues, and its expression was associated with HCC. The expression of EPDR1 was associated with advanced stages of HCC [25]. In contrast, EPDR1 exerts inhibitory effects in ovarian cancer and BC. Zhao et al. confirmed that EPDR1 was downregulated in epithelial ovarian cancer (EOC) tissues and its overexpression significantly inhibited the proliferation, migration, and invasion of EOC cells and associated with the inhibition of PI3K/AKT signaling pathway [26]. Liu et al. verified the similar oncogenic role of EPDR1 in ovarian cancer [27]. Liang et al. concluded that EPDR1 was lowly expressed in BC tissues and cells, and its overexpression suppressed proliferation, invasion and promoted apoptosis in BC cells, probably due to its increased expression of p53, p21, and Bcl-2 and inhibition of Bax expression [28]. In this study, overexpression of EPDR1 inhibited proliferation, migration, and invasion of BC cells, which is consistent with the results of Liang et al. [28]. In addition, we demonstrated that overexpression of EPDR1 down-regulated the expression of interstitial-related proteins in BC cells and inhibited tumor growth in vivo. These results suggest that EPDR1 may inhibit EMT in breast cancer. However, our study only examined the effect of EPDR1 expression on the expression of interstitial-related proteins in breast cancer, and comprehensive studies are needed to confirm the direct link between EPDR1 overexpression and EMT inhibition.

MicroRNAs (miRNAs) are a category of non-coding RNAs, typically about 22 nucleotides long, that suppress gene expression at the post-transcriptional stage. They achieve this by binding to the 3’-untranslated region of their target messenger RNAs (mRNAs) [29]. miRNAs can function either as oncogenes or as tumor suppressors by regulating core mRNAs and signaling pathways [30]. The study suggested that miRNAs may be regulated by the tumor microenvironment, including hypoxia, and are involved in regulating hypoxia-induced responses [31]. Interestingly, EPDR1 expression is reduced in hypoxic BC cells and several studies have shown that its expression is regulated by miRNAs [26, 27]. In this study, it was hypothesized that the reduction of EPDR1 might be associated with hypoxia-induced miRNAs. Therefore, we analyzed those differentially expressed genes in the GSE49999 dataset containing three hypoxic BC MCF-7 cells and three normoxic BC MCF-7 cells. The results showed that miR-181a-5p expression was upregulated in hypoxic BC cells. Our findings also demonstrated a potential binding site for miR-181a-5p to EPDR1 mRNA, which was highly expressed in BC tissues, and its high expression was associated with lower overall survival in BC patients. miR-181a-5p expression was upregulated by cisplatin treatment in TNBC cells, and cell apoptosis was induced in TNBC through inhibition of Bcl-2 reported by Rania et al. [32]. Liu et al. found that miR-181a-5p promoted development of BC cells through inhibiting TUSC3 expression [33]. Similarly, Zhai et al. demonstrated that miR-181a-5p promoted the proliferation and invasion of BC cells, and also improved cell glycolysis in BC cells through the activation of the PTEN/AKT pathway by targeting NDRG2 [34]. Nevertheless, several studies have reported the oncogenic effects of miR-181a-5p in BC. Liu et al. found that overexpression of miR-181a-5p inhibited BC cell proliferation and invasion by targeting KLF6 and KLF15 expression [35]. Our study confirmed that hypoxia inhibited EPDR1 expression in BC cells through upregulation of miR-181a-5p. Interestingly, hypoxia-upregulated miR-181a-5p reversed the inhibitory effects of Epirubicin treatment on BC cell proliferation, migration, invasion, and the expression of interstitial-related proteins through inhibiting EPDR1.

Meanwhile, we screened genes associated with EPDR1 expression in BC using bioinformatics and demonstrated that TRPC1 expression was positively correlated with EPDR1. TRPC1 levels of BC tissues was associated with high overall survival in BC patients. TRPC1 is a member of the transient receptor potential (TRP) channel superfamily and interacts with STIM proteins [36]. Zhang et al. found that TRPC1 inhibited proliferation, migration, invasion, and EMT of MCF-7 cells through inhibition of PI3K/AKT signaling pathway [18]. Zhao et al. reported that EPDR1 inhibited ovarian cancer cell proliferation, invasion, and migration through the PI3K/AKT pathway [26]. PI3K/AKT also stimulated tumorigenesis and progression [37]. PI3K/AKT pathway was reported to induce epirubicin resistance in BC cells [19]. In this study, TRPC1 expression was downregulated in epirubicin-resistant BC cells, while the PI3K/AKT signaling pathway was activated in epirubicin-resistant BC cells. Overexpression of EPDR1 inhibited PI3K/AKT signaling pathway activation in Epirubicin-resistant BC cells, and silencing TRPC1 reversed the inhibitory effect of EPDR1 overexpression on PI3K/AKT signaling pathway in epirubicin-resistant BC cells. Furthermore, our results demonstrated that EPDR1/TRPC1 inhibited the proliferation, migration, invasion, and interstitial-related protein expression of epirubicin-treated resistant BC cells by suppressing PI3K/AKT signaling pathway and increased the sensitivity of epirubicin-resistant BC cells.

In conclusion, hypoxia may reduce the sensitivity of BC cells to epirubicin by down-regulating EPDR1 expression in BC cells. Mechanistically, hypoxia may inhibit EPDR1 expression through up-regulation of miR-181a-5p. EPDR1 may inhibit the PI3K/AKT pathway dependent on up-regulation of TRPC1, reducing the sensitivity of BC cells to epirubicin. Collectively, hypoxia-induced miR-181a-5p may reduce the epirubicin sensitivity through inhibiting the EPDR1/TRPC1 axis in BC cell. Our results indicate that hypoxia-induced miR-181a-5p may reduce epirubicin sensitivity in BC cells via inhibiting the activation of the PI3K/AKT signaling pathway. This study provides a theoretical basis for the application of epirubicin in the treatment of BC. However, we only performed an in vitro experiment to demonstrate that the EPDR1 down-regulation induced by hypoxia affects the sensitivity of epirubicin in BC cells. The mechanism identified in this study needs to be further investigated in the animal model. Furthermore, this study only detected the regulatory effect of EPDR1 overexpression on TRPC1 expression, and the detailed molecular mechanism of its regulation of TRPC1 expression remains to be further analyzed in future studies.

## Data Availability

The datasets used and/or analyzed during the current study are available from the corresponding author on reasonable request.
